# Aid alignment: a longer term lens on trends in development assistance for health in Uganda

**DOI:** 10.1186/1744-8603-9-7

**Published:** 2013-02-20

**Authors:** Elizabeth Stierman, Freddie Ssengooba, Sara Bennett

**Affiliations:** 1 Primary author, P.O. Box 38086, Lusaka, Zambia; 2 Makerere University School of Public Health, MaKSPH Bld, Mulago Hill Road, P.O. Box 7072, Kampala, Uganda; 3 Johns Hopkins Bloomberg School of Public Health, 615 North Wolfe St, Baltimore, MD 21205, USA

**Keywords:** Foreign aid, International cooperation, Health policy, Health financing, Sector-wide approach, Paris declaration

## Abstract

**Background:**

Over the past decade, development assistance for health (DAH) in Uganda has increased dramatically, surpassing the government’s own expenditures on health. Yet primary health care and other priorities identified in Uganda’s health sector strategic plan remain underfunded.

**Methods:**

Using data available from the Creditor Reporting System (CRS), National Health Accounts (NHA), and government financial reports, we examined trends in how donors channel DAH and the extent to which DAH is aligned with sector priorities. The study follows the flow of DAH from the donor to the implementing organization, specifying the modality used for disbursing funds and categorizing funds based on program area or support function.

**Findings:**

Despite efforts to improve alignment through the formation of a sector-wide approach (SWAp) for health in 1999 and the creation of a fund to pool resources for identified priorities, increasingly DAH is provided as short-term, project-based support for disease-specific initiatives, in particular HIV/AIDS.

**Conclusion:**

These findings highlight the need to better align external resources with country priorities and refocus attention on longer-term sector-wide objectives.

## Background

The 2005 Paris Declaration spelled out five key principles of aid effectiveness—ownership, alignment, harmonization, results, and mutual accountability [1]—which have received further support through subsequent meetings in Accra (2008) and Busan (2011). Within the health sector, the launch of the International Health Partnership (IHP+) in 2007 increased the global emphasis upon these principles, encouraging development partners to align assistance with national health plans and use country systems to disburse and manage aid.

These global agreements built upon earlier efforts initiated in the mid-1990s to bring together government, donors and other key stakeholders to jointly define sector priorities and develop a common strategy to achieve those goals [2-9]. Known as a Sector Wide Approach (SWAp), this process promoted the use of common arrangements for planning, budgeting, financing, managing, monitoring, and evaluating sector-wide investments over the medium and long-term. Since 1997, more than 30 countries in Africa, Latin America, Asia, and the Pacific have adopted a SWAp for health [8,9].

Yet despite increasing efforts to better align development assistance, with the exception of a few studies that have focused largely on the global level [10-13], there has been little research that systematically measures, in quantitative terms, trends in the flow of external resources for health, or the extent to which these resources are aligned with country health priorities. A recent joint independent evaluation of the Paris Declaration found that while the Declaration has strengthened agreed norms and standards for better practice in development assistance, progress on aid alignment was uneven [14]. The evaluation recommended that policymakers in donor countries “match the crucial global stakes in aid and reform with better delivery on promises made”. This leads us to ask, to what extent has the rhetoric regarding aid alignment in the health sector, matched on the ground realities?

Using Uganda as a case study, this paper explores trends in development assistance for health (DAH) from 1999 to 2009 in the context of efforts to better coordinate assistance, enhance country ownership, and align external resources with national health priorities. Uganda was chosen for study because it was an early adopter of strategies to better coordinate DAH [6,8]. Additionally, with a relatively stable macroeconomic and political environment [15,16], a relatively sound public financial management system [15,17], the initial commitment from key government leaders [15,18,19] and friendly relations with the donor community [15,20], conditions in Uganda represent those in which donor coordination mechanisms would be expected to function well [8,21].

### The Ugandan context

In 1997, the Government of Uganda (GOU), with its international partners, developed a national plan to fight poverty and ill health within the country. The Poverty Eradication Action Plan (PEAP) laid the groundwork for the creation of the Poverty Action Fund (PAF) in 1998, which was designed to bring together donor resources, debt relief savings from the Highly Indebted Poor Country (HIPC) initiative and government funds into a common basket to finance priority programs. Within the health sector, the Ministry of Health (MOH) and development partners adopted a SWAp to better coordinate health projects and target efforts towards realizing objectives articulated within the Health Sector Strategic Plan (HSSP). Central to the plan was a renewed focus on primary health care: the plan describes the GOU’s strategy to deliver a core package of preventative and basic curative health services—the Ugandan National Minimum Health Care Package (UNMHCP)—through a decentralized health system designed to bring services closer to the people [22].

Donors were encouraged to help finance the HSSP through budget support, provided either as general contributions to the government budget or channeled through the PAF. The shift away from project-based support towards budget support aimed to promote greater country ownership and was viewed to be more efficient and more supportive of the longer-term, broad health sector objectives envisioned within the SWAp [23-25]. Many donors did adopt this approach; within the first few years of the SWAp, the number of donors providing budget support increased from five in 2000/01 to twelve in 2002/03 [25].

Where donors chose to continue providing project-based support rather than budget support, they were encouraged to plan projects in coordination with the overall SWAp and record project expenditures on the Medium Term Expenditure Framework (MTEF), which sought to incorporate public expenditures by both the GOU and donors within a single three-year budget framework. This approach aimed to improve overall planning and make projects less disruptive to the health system by improving predictability of funds and better aligning project-based support with the HSSP [24].

However, while there have been consistent efforts by the GOU to promote alignment of DAH with government goals and to encourage budget support by donors, these efforts may have been undermined by concerns about corruption. The World Bank’s aggregated indicator on the control of corruption shows a slight negative trend throughout the 2000s [26]. The perception of corruption as being as being a serious challenge to Uganda’s health sector was aggravated by allegations of misuse of funds from the Global Fund to Fight AIDS, Tuberculosis and Malaria (GFATM) that surfaced in 2005 and led to the temporary suspension of GFATM support to Uganda and, recently in 2011, by allegations of the embezzlement of donor funds intended for post-conflict reconstruction in northern Uganda.

## Methods

### Objectives

This paper aims to:

•Analyze trends over time in how donors have channeled DAH and the extent to which there has been a shift towards on-budget and pooled funding for health; and

•Assess the degree to which the allocation of DAH is aligned with country priorities as articulated in the HSSP.

### Key terms and conceptual framework

For the purpose of this study, development assistance for health (DAH) is defined as official development assistance for health sector activities, including disease-specific and general health sector support. This includes both grants and concessional loans provided by governments, multilateral institutions, and official agencies^a^ as reported within the Organization for Economic Co-operation and Development’s (OECD) Creditor Reporting System (CRS) [27]. Debt relief savings and donations by private individuals and foundations (e.g. Bill and Melinda Gates Foundation) are excluded from the analysis. To the extent possible, DAH is based on actual disbursements as reported by donors within the CRS or as recorded on GOU financial records, rather than commitments.

This study utilizes a health financing framework based on the approach used in National Health Accounts (NHA) in which resources are tracked from funding source to financing agent, and from financing agent to service provider or function. This paper adapts this framework to follow how donors (the funding source) channel resources to implementing organizations (the financing agent), specifying the modality used by donors for planning, pooling, and disbursing funds: general budget support (GBS), sector budget support to the PAF (SBS-PAF), on-budget project support, or off-budget project support. Resources are then categorized based on the relevant program area or support system (the function). For this study, nine categories are identified based on the priority program areas and support systems identified within the UNMHCP [22] (Table 1).

**Table 1:** Health financing framework and definitions

**Source of funds**	**Modality for planning, pooling, and disbursing funds**	**Program area or support system**
**Governments**	**General budget support (GBS):** GBS is provided centrally to the GOU and pooled with domestic resources. Funds are not earmarked for specific sectors. Instead they are allocated to health and other sectors based upon the GOU’s general budgeting process and approved by Parliament. Funds are disbursed and managed through the public financial management system.	HIV/AIDS and sexually transmitted diseases (STDS)
		Malaria
	**Sector budget support to PAF (SBS-PAF):** The Poverty Action Fund (PAF) pools resources from donors, the government, and debt relief savings into a single basket for priority development activities in support of the PEAP. Donors can contribute to the PAF through budget support specifically earmarked for the health sector or through general contributions to the PAF budget, of which a proportion is allocated towards priority health programs. All funds are managed through the public financial management system.	
		Tuberculosis
**Multilateral Institutions**		Essential clinical care, management of childhood illness and mental health
• World Bank		
• Africa Development Bank		
• United Nations		
	**On-budget project support:** On-budget project support is planned in consultation with the GOU and other donors through the MTEF. Funds are designated for specific projects in the public sector, which may be implemented by the GOU, donor government agencies, or multilateral organizations. The process aims to better align external funds with government priorities and avoid duplication. However, unlike budget support, the decision on what type of projects will be funded and how funds will be managed is ultimately the decision of the donor who finances the project.	
		Sexual and reproductive health
		Public health interventions
		• Environmental health
		• Disease eradication
**Official Agencies**		
		• Immunization
• Global Fund for AIDS, Tuberculosis and Malaria (GFATM)		
	**Off-budget project support:** Off-budget project support is not planned within the MTEF. It includes projects within the private sector, as well as some projects implemented by donors and multilateral organizations but not planned through the MTEF. With this approach, donors determine the type of projects that will be funded and the timetable for implementation of activities. This approach may be selected in order to avoid macroeconomic budget ceilings, to conform to guidelines of donor countries, to strengthen civil society, or because of the belief that government agencies are inefficient or corrupt. This funding approach utilizes financial management systems outside the existing government structures and may create parallel implementation units.	
• Global Alliance for Vaccines and Immunizations (GAVI)		
		Nutrition
		Health education and school health
		Support systems
		• Personnel development
		• Infrastructure
		• Administration

The modality used to disburse DAH has important implications for the alignment of donor funds with country priorities. Since budget support is pooled with general government revenue and allocated, disbursed, managed and accounted for using country budgeting processes and financial management systems, it provides recipient governments the greatest control and ownership; as such, this modality would be expected to align closely with the government’s stated priorities. Meanwhile the extent to which project-based support is aligned with country priorities varies depending on the donor’s engagement in country coordination mechanisms, as well as the extent to which donor priorities match country priorities. Project support planned in coordination with the SWAp and recorded on the government’s financial planning framework, the MTEF, would be expected to align more closely than project support planned outside these coordination mechanisms.

This study measures alignment based on the extent to which donor resources are allocated towards country-identified health priorities, specifically the core program areas and support systems which comprise the UNMHCP. The HSSP estimated the costs of delivering the UNMHCP over a five-year period at 954 million USD, with the majority of funds (65%) allocated towards improving support systems, including basic health infrastructure and personnel development. Most of the remaining budget (21%) was dedicated to providing basic health care, which includes the three UNMHCP program areas of essential clinical care, management of childhood illness, and mental health services. A smaller proportion of the budget was to support sexual and reproductive health care, public health interventions, HIV/AIDS, malaria, tuberculosis, nutrition, health education and school health [22].

### Data sources

**Figure 1:**
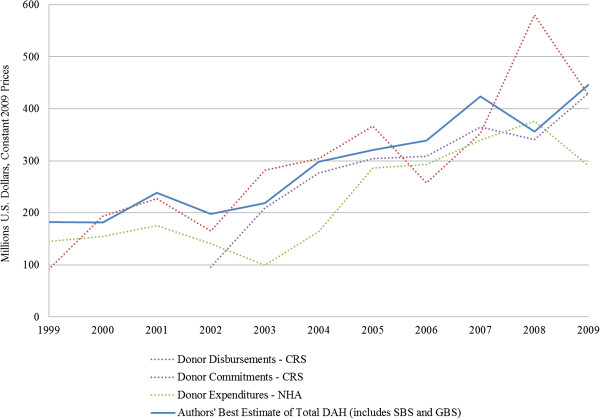
**Trends in total DAH 1999–2009.** Sources: WHO [29]; OECD/DAC [27].

Data on DAH was collected from the CRS, NHA, and GOU financial reports, including Annual Budget Performance Reports (ABPR), Approved Budget Estimates, Background to the Budget (BTTB) Reports, and Annual Health Sector Performance Reports (AHSPR) [27-44]. Based on a comparison of available data for the study period 1999 to 2009, CRS data was determined to be the most comprehensive source of information on external funding for health for 2003 onward, but NHA data was found to provide better information prior to 2003 (Figure 1).

The authors’ estimates of total DAH are based upon either NHA expenditures or CRS disbursements, depending on the year, plus an estimate of the share of the government’s health budget financed through budget support, both GBS and SBS-PAF (Additional file 1). On-budget project support is the amount of project support recorded on the MTEF. Off-budget project support was estimated by subtracting the amount of on-budget project support from the amount of total project support reported within NHA or the CRS. The amount of GBS provided to the health sector was estimated by multiplying total GBS by the proportion of the government’s budget allocated for health. SBS-PAF was calculated in a similar manner: multiplying total donor contributions to the PAF by the proportion of the PAF budget allocated for health. Donor contributions include both earmarked and non-earmarked grants. Since some grants earmarked for health are already included within CRS estimates, these funds were subtracted from the authors’ estimate of total DAH to avoid double-counting.

All estimates were converted to 2009 constant U.S. dollars. The CRS converts ODA from current to constant U.S. dollars using a deflator to adjust for changes in the exchange rate and inflation in the currency in which the flow occurred between the year of the flow and the base year [45]. The same approach was used to convert estimates reported in current Ugandan shillings to constant U.S. dollars. First, Ugandan shillings for the relevant year were converted to current U.S. dollars using the Bank of Uganda’s (BOU) official exchange rate for the year. Current U.S. dollars were then converted to constant U.S. dollars by dividing this amount by the deflator used by CRS for “total DAC flows”—the average of the deflators of individual Development Assistance Committee (DAC) donors, weighted by each donor’s total ODA [45].

Estimates are presented for the calendar year, consistent with the reporting of CRS data. Estimates reported based on the GOU fiscal year have been attributed to the calendar year which corresponds to the later fiscal year (e.g. funds for FY 2005/06 have been attributed to the 2006 calendar year).

## Results

### Trends in how DAH is channeled

**Figure 2:**
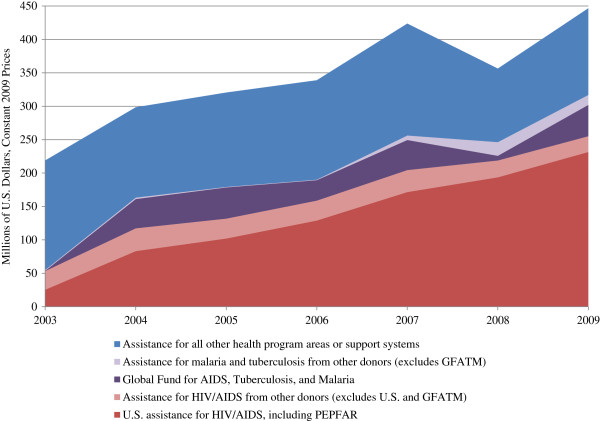
**Trends in funding for disease-specific initiatives.** Sources: OECD/DAC [27].

Total DAH has increased dramatically since 1999 (Figure 1). From estimates of 180–240 million USD between 1999 and 2003, we see a sharp increase in DAH starting in 2004 with disbursements rapidly increasing to over 420 million USD by 2007. This has been driven in large part by the launch of several global health initiatives, such as the GFATM and in particular the President’s Emergency Plan for AIDS Relief (PEPFAR) (Figure 2). In 2009, USG assistance for HIV/AIDS, including PEPFAR, amounted to 232 million USD, over half of all DAH for the year. GFATM, the second largest donor in 2009, provided an additional 47 million USD to finance HIV/AIDS, malaria and tuberculosis programs.

**Figure 3:**
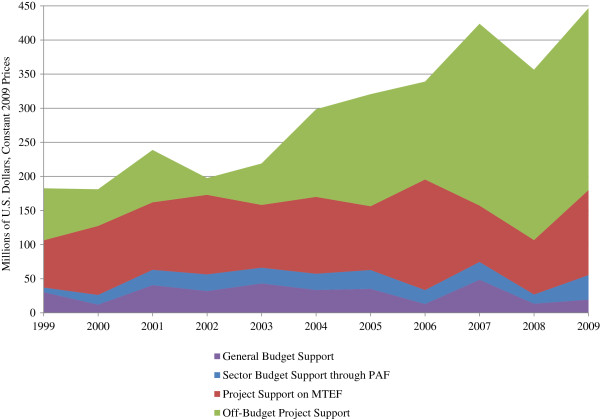
**Budget mechanisms used to channel DAH.** Sources: MOH [28,39-43]; WHO [29]; MOFPED [30-38]; OECD/DAC [27].

Much of the growth in DAH since 2003 has been due to increases in project-based funding, of which most is planned outside of the government’s financial planning framework (Figure 3). Based on the authors’ estimates^b^, more than half of DAH in recent years was provided as off-budget, project-based funds. Similarly, the MOH estimates that 41% of donor project support in FY 2006/07 and 59% in FY 2009/10 was off-budget [41]. The large amount of off-budget project support can largely be explained by the non-participation of PEPFAR and other USG projects in national planning frameworks like the MTEF.

**Figure 4:**
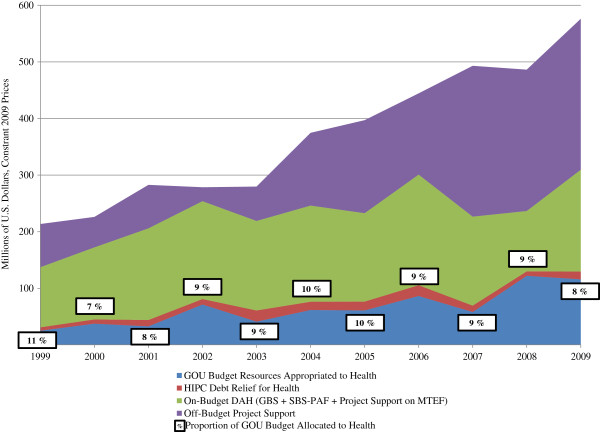
**Trends in total health expenditures 1999–2009.** Sources: MOH [28,39-43]; WHO [29]; MOFPED [30-38]; OECD/DAC [27]; Uganda Debt Network [44].

In comparison to appropriations by the national government, donors provided 3–7 times more resources to the health sector over the study period (Figure 4). GOU resources for health have steadily risen from 25.1 million USD in 1999 to 115.8 million USD in 2009, and have remained a relatively constant proportion of the total government budget (8-11%). However, this amount remains small relative to rising levels of DAH.

**Figure 5:**
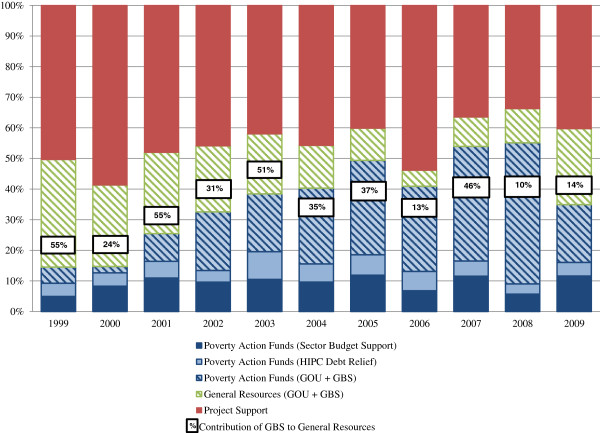
**Sources of funds for GOU health budget.** Sources: MOH [28,39-43]; WHO [29]; MOFPED [30-38]; OECD/DAC [27]; Uganda Debt Network [44].

Donors contribute more than half of funds recorded on the GOU’s official health budget through a combination of project support, GBS, and SBS-PAF (Figure 5). Project-based support from donors is consistently a large source of funds. Throughout the study period (1999–2009), project-based support contributed to 34-59% of the official health budget.

The PAF is another important mechanism for financing the government’s health budget. Since its creation in 1998, the PAF has become an increasingly important source of funds; in recent years, 35-54% of the government’s health budget was financed through the PAF. However, budget support to the PAF has fallen as a proportion of total PAF funds. Donors provided approximately half of PAF funds in FY 1999/2000 and HIPC savings made up much of the rest [28]. In contrast, in FY 2007/08 donor support provided approximately 11% and savings from HIPC only 6% of the total PAF budget [34]. Contributions from the GOU’s own resources and, to a limited degree, donors through GBS now provide the main source of PAF funds [34].

### Alignment of funds with sector priorities

**Figure 6:**
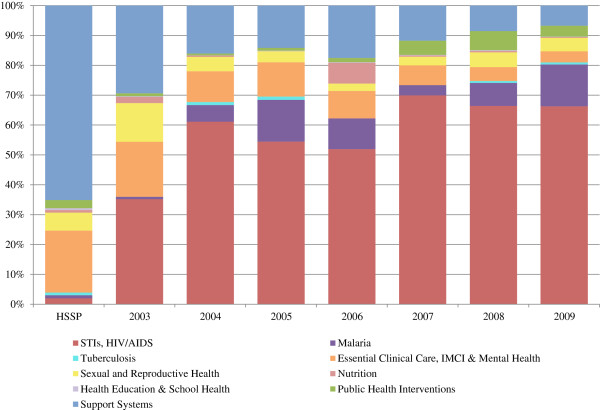
**Allocation of project support by Uganda National Minimum Health Care Package (UNMHCP) cost category.** Sources: OECD/DAC [27].

Since 2003, we observe a large increase in DAH earmarked for specific diseases, in particular HIV/AIDS (Figure 2). In recent years, more than three-quarters of DAH was allocated towards HIV/AIDS, malaria, and tuberculosis. This is in marked contrast to the blueprint set forth in the HSSP for allocating resources amongst the package of priority health programs and support systems. The proposed five-year budget in the HSSP allocates only 2% of funds towards HIV/AIDS, 1% to malaria, and 1% to tuberculosis^b^[22] (Figure 6).

Examining the distribution of project support^c^ by UNMHCP cost category, we observe that funding for support systems (infrastructure, personnel development, administration) has steadily declined from 29% of project support in 2003 to 7% in 2009—much lower than the 65% of funds allocated towards this category within the budget proposed in the HSSP. Similarly, project support for basic health care (essential clinical care, management of childhood illness, and mental health) has declined from the 18% of funds in 2003 to 4% in 2009—considerably less than the 21% proposed allocation. Meanwhile funding for public health interventions (immunization, disease eradication, environmental health) has increased with the introduction of the Global Alliance for Vaccines and Immunizations (GAVI) in 2007, from approximately 1% of the budget in 2006 to an estimated 4% of the budget in 2009—comparable with the proposed allocation of 3%.

Examining these trends in closer detail based on project information available through CRS microdata and official GOU budget estimates, we observe important differences in the main sources of funding for different program areas. Funding for health support systems and essential clinical care and management of childhood illnesses is provided through numerous, small projects (generally less than 1 million USD annually) from a diverse group of donors. Meanwhile, funding for HIV/AIDS, malaria, tuberculosis, and immunizations is dominated by multimillion dollar global health initiatives, namely PEPFAR, GFATM, and GAVI.

## Discussion

While the issue of improving donor coordination and alignment continues to be a stated priority for the development community, in Uganda the proportion of DAH provided off-budget has increased in the past decade as the proportion provided as GBS and SBS-PAF has declined. While initially the PAF was effective in raising external funds for identified health priorities and channeling greater resources to districts, the PAF has become obsolete as a mechanism for donor coordination; we estimate that only 4% of DAH in 2008 and 8% in 2009 was channeled as SBS-PAF. Similarly, we estimate that, for those same years, only 4% of DAH was channeled as GBS. As a proxy for coordination and alignment, the reduction in donor contributions to the PAF and general budget indicates an off-track course in coordinated and effective aid.

As a result, despite the increase in DAH, funding for the UNMHCP continues to fall short of targets. In FY 2008/09, of the 41.2 USD per capita required to fully fund the package, the budget provided only 12.50 USD [46]. This financing gap has immediate impacts on quality of care: as of November 2008, half of government health staff posts were vacant and shortages were even more pronounced in lower level units [46]. Additionally, construction of many health facilities remains incomplete while others are in need of rehabilitation. The rise of vertical, disease-specific initiatives appears to have complicated efforts to bring together partners under a common sector-wide strategy [10,11,19,25,47-49]. In Uganda, both PEPFAR and GFATM created parallel systems of project and financial management, with separate monitoring and reporting requirements and, in the case of PEPFAR, a separate funding and audit timetable [25]. These were viewed as out of sync with the objectives of the SWAp and, in some ways, disruptive: requiring additional time and efforts to respond to the separate requirements of different projects and diverting limited human resources [25,50]. Additionally, in the context of macroeconomic budget ceilings, project-based support may crowd out funding for other health sector activities [16,19,25,51,52].

While DAH in Uganda appears to be poorly aligned with the budget priorities set out in the HSSP, there may be a case that investment in the control and treatment of priority diseases such as STIs, HIV/AIDS and TB can simultaneously strengthen broader health systems. The empirical question of the extent to which HIV/AIDS-specific services do indeed build health systems remains largely unanswered [53,54]; studies in Uganda indicate mixed results [55,56]. However, even if we allow that some of the funding for HIV/AIDS in Uganda has indeed served to strengthen health systems, it would seem that there remains a substantial gap between the need for systems strengthening identified by the government and investment in this area by donors.

The emphasis among select donors to channel funds as project-based support, concentrating on short-term projects that can achieve measureable results by the end of the project period has contributed to the preference for contracting services through private firms and NGOs in the case of PEPFAR, or to establish parallel implementation units within the government in the case of GFATM, rather than providing budget support using existing government structures [50]. Qualitative research illuminates some of the reasons behind this preference, namely the perception that the use of existing government structures would delay timely disbursements and implementation of activities and concerns about financial management [25,50]. However, this approach focused on demonstrating short-term results generally fails to consider the importance of longer-term investments in health infrastructure, personnel development, or institutional capacity-building within the government.

The time trends depicted in the figures reflect a gradually worsening situation with respect to the proportion of general reserves financed through budget support, and gradually rising levels of off-budget support. While it is tempting to attribute these trends largely to the nature of donor engagement, and in particular the establishment of PEPFAR and the GFATM in 2003/04, there may be other explanations. For example, there are factors on the recipient side which have perhaps undermined the ability of Uganda to attract flexible DAH. As noted in the introduction, corruption has been a concern for donors in the Uganda context and this was exacerbated by high profile events in 2005 and 2011. Even donors such as the UK Department for International Development that strongly favor budget support have delayed disbursements due to dissatisfaction with financial management in Uganda, and due to political issues [57]. In addition some analysts have suggested that leadership in Uganda’s health sector in recent years has been weak compared to other sectors [58].

### Limitations

Data limitations in conducting this study are acknowledged: there were inconsistencies within and between data from the CRS, NHA, and GOU financial reports [10,11,19]. While the CRS reports figures in constant and current U.S. dollars for the calendar year, GOU and NHA reports figures in current Ugandan shillings for the fiscal year. When comparing between sources, figures were converted to constant dollars using official exchange rates, but it should be noted that exchange rates were highly variable for Uganda during the study period. Additionally, despite efforts to encourage donors to accurately report spending, much spending still goes unreported. Nonetheless, it is likely that off-budget funds are most likely to be under-reported by donors, suggesting that our findings may be biased towards underestimating off-budget DAH.

## Conclusions

The mismatch between the allocation of DAH and country needs (based on patterns of disease burden) or country priorities (based on health sector strategic plans) has been previously observed [59-61]. In particular, it has been argued that donor spending on HIV/AIDS appears to be in excess of need [59]. This issue and its implications for country ownership of donor programs and aid alignment was one of the catalytic factors behind the development of the IHP+ with its emphasis on reciprocal accountability, reducing the fragmentation of aid for health, and promoting alignment with country priorities [62]. The same set of concerns has also influenced President Obama’s Global Health Initiative (GHI) which prioritizes building upon and expanding existing country owned platforms, and strengthening health systems [63]. As earlier research demonstrated the potential of SWAps in promoting aid alignment and country ownership, it may be tempting to infer that the increased international policy focus upon these issues, as embodied by the IHP+ and President Obama’s GHI, has led to greater alignment. Sadly, in Uganda at least, this is not the case.

This study is one of the first to provide a detailed country-specific picture of patterns of DAH and how they have evolved over time. The picture is not a salutary one: despite the existence of the Ugandan PAF and a health SWAp, DAH in Uganda is highly fragmented and often off-budget. Most DAH is provided as support to short-term projects rather than sector programs planned over the longer term. This pattern has become more marked over time. While our study only contains data up to 2009, there is no evidence to suggest that there has been a major shift in patterns of DAH since that time.

While Uganda was initially a leader in the development of strategies to better coordinate donor assistance for health, during the past decade, problems of corruption, financial management, and relatively weak leadership within the health sector may have attenuated the Ugandan government’s ability to encourage donors to provide flexible and aligned aid to the health sector. It is impossible to disaggregate the relative significance of donor factors versus recipient factors in terms of explaining the trends observed, or to predict what might have happened if such recipient-related factors had not arisen. However, it is not clear if the preference to mitigate risks through the use of more rigid, donor-controlled funding mechanisms is the most appropriate way to build country systems and institutions.

A sizeable proportion of the DAH received by Uganda is already specified for particular diseases; this leaves little room for an aid-dependent government to gather additional support from donors to finance nationally-determined health sector priorities. There is a need to acknowledge the limitations of donor coordination mechanisms in Uganda, and seek more fundamental reform in how donors plan, budget, and finance DAH so that reality aligns better with the rhetoric.

A significant barrier to efforts to harmonize and align aid lies in the way in which donors make decisions regarding the allocation of DAH. Within the United States, for example, most high-level funding decisions take place in budget committees and congressional offices, far removed from the health centers and communities where funds are ultimately destined. Presidential initiatives and congressional earmarks may crowd out aid that is more relevant and responsive to the needs of the local community and restrict investments in sustainable institutions and health systems [64]. Particularly in the context of the current recession, there is a clear tension between the desire to earmark funds for politically popular interventions, versus ensuring that DAH is used as efficiently as possible, to support country health goals. While donor governments clearly need to further explore and address the obstacles which they face to making flexible and aligned aid available, these efforts also need to be matched by stronger leadership and financial management on the part of recipient governments.

## Endnotes

^a^Official agencies include the Global Fund to Fight AIDS, Tuberculosis and Malaria (GFATM) and the Global Alliance for Vaccines and Immunization (GAVI).

^b^It should be noted that the small percentages allocated towards HIV/AIDS, malaria, and tuberculosis in the HSSP budget are not intended to reflect the full costs of treating these diseases. Budget allocations to other cost categories (e.g. infrastructure, essential clinical care, and child health) also support prevention and treatment of these diseases.

^c^Budget support is excluded from the analysis as support is not earmarked for specific program areas. Rather it is pooled with government resources and allocated to program areas based on government budgeting processes.

## Competing interests

The authors declare that they have no competing interests.

## Authors’ contributions

ES and SB made substantial contributions to the conception and design of the study. ES took the lead in compiling and analyzing the data, with contributions from SB and FS in interpreting the data. ES took the lead in writing the first draft of the manuscript and SB and FS critically reviewed it. All authors have given final approval of the version to be published.

## Supplementary Material

Additional file 1 DAH data used in analysis. Click here for file

## References

[B1] Organization for Economic Co-operation and Development Paris Declaration on Aid Effectiveness: Ownership, Harmonization, Alignment, Results, and Mutual Accountability. The Second High Level Forum on Aid Effectiveness 2005 Paris

[B2] Cassels A Janovsky K Better health in developing countries: are sector-wide approaches the way of the future? Lancet 1998 352 9142 1777 1779 10.1016/S0140-6736(98)05350-1 9848371

[B3] Buse K Keeping a tight grip on the reins: donor control over aid coordination and management in Bangladesh Health Policy Plan 1999 14 3 219 228 10.1093/heapol/14.3.219 10621239

[B4] Foster M Brown A Conway T Sector-wide Approaches for Health Development: A Review of Experience 2000 Geneva: World Health Organization WHO/GPE/00.1

[B5] Hutton G Tanner M The sector-wide approach: a blessing for public health? Bull World Health Organ 2004 82 893 894 15654401 PMC2623098

[B6] Jeppsson A SWAp dynamics in a decentralized context: experiences from Uganda Soc Sci Med 2002 55 2053 2060 10.1016/S0277-9536(01)00345-8 12406470

[B7] Swedish International Development Cooperation Agency Mapping of Sector-wide Approaches in Health 2003 London: Institute for Health Sector Development

[B8] Bank W Education and Health in Sub-Saharan Africa: A Review of Sector-wide Approaches 2001 Washington: World Bank

[B9] Vaillancourt D Do Health Sector-wide Approaches Achieve Results? Emerging Evidence and Lessons from Six Countries. IEG Working Paper 2009/4 2009 Washington: World Bank

[B10] Ravishankar N Gubbins P Cooley RJ Leach-Kemon K Michaud CM Jamison DT Financing of global health: tracking development assistance for health from 1990 to 2007 Lancet 2009 373 9681 2113 2124 10.1016/S0140-6736(09)60881-3 19541038

[B11] Sridhar D Batniji R Misfinancing global health: a case for transparency in disbursements and decision making Lancet 2008 372 9644 1185 1191 10.1016/S0140-6736(08)61485-3 18926279

[B12] Michaud C **Development assistance for health (DAH): recent trends and resource allocation** Paper Prepared for Second Consultation Commission on Macroeconomics and Health 29–30 October 2003 Geneva: World Health Organization

[B13] Piva P Dodd R Where did all the aid go? An in-depth analysis of increased health aid flows over the past 10 years Bull World Health Organ 2009 87 930 939 10.2471/BLT.08.058677 20454484 PMC2789359

[B14] Wood B Betts J Etta F Gayfer J Kabell D Ngwira N Sagasti F Samaranayake M The Evaluation of the Paris Declaration Final Report 2011 Copenhagen: Danish Institute for International Studies

[B15] Lister S Baryabanoha W Steffensen J Williamson T Evaluation of General Budget Support: Uganda Country Report 2006 Birmingham: University of Birmingham

[B16] Williamson J Putting Aid on Budget: A Case Study of Uganda. A Study for the Collaborative Africa Budget Reform Initiative and Strategic Partnership with Africa 2008 Oxford: Mokoro Ltd

[B17] Country Policy and Institutional Assessment (CPIA) for Sub-Saharan Africa 2011 http://datatopics.worldbank.org/cpia/country/uganda, Accessed 10 April 2012

[B18] Zambara S Tashobya CK, Ssengooba F, Oliveira Cruz V **Foreword** Health Systems Reforms in Uganda: Processes and Outputs 2006 London: London School of Hygiene & Tropical Medicine

[B19] Örtendahl C The Uganda Health SWAp: New Approaches for a More Balanced Aid Architecture? 2007 London: HLSP Institute

[B20] Hauser E Ugandan relations with Western donors in the 1990s: what impact on democratisation J Mod Afr Stud 1999 37 4 621 641 10.1017/S0022278X9900316X

[B21] Lake S Musumali C Zambia: The role of aid management in sustaining visionary reform Health Policy Plan 1999 14 3 254 263 10.1093/heapol/14.3.254 10621242

[B22] Ministry of Health Health Sector Strategic Plan 2000/01-2004/05 2000 Kampala: Ministry of Health

[B23] Oliveira Cruz V Cooper R McPake B Yates R Ssengooba F Omaswa F Tashobya CK Murindwa G Tashobya CK, Ssengooba F, Oliveira Cruz V Is the Sector-Wide Approach (SWAp) improving health sector performance in Uganda Health Systems Reforms in Uganda: Processes and Outputs 2006 London: London School of Hygiene & Tropical Medicine 29 44

[B24] Ssengooba F Yates R Oliveira Cruz V Tashobya CK Tashobya CK, Ssengooba F, Oliveira Cruz V Have systems reforms resulted in a more efficient and equitable distribution of resources in the Ugandan health sector Health Systems Reforms in Uganda: Processes and Outputs 2006 London: London School of Hygiene & Tropical Medicine 109 120

[B25] Oliveira Cruz MP Global Health Initiatives and aid effectiveness: insights from a Ugandan case study Glob Heal 2011 7 20 10.1186/1744-8603-7-20 PMC3148970 21726431

[B26] Worldwide Governance Indicators Country Data Report for Uganda, 19967–2011 2010 http://info.worldbank.org/governance/wgi/pdf/c225.pdf, Accessed 10 April 2012

[B27] Organization for Economic Co-operation and Development Development and Cooperation Development Assistance Committee: Creditor Reporting System 2011 http://stats.oecd.org/Index.aspx?datasetcode=CRS1

[B28] Ministry of Health Financing Health Services in Uganda 1998/1999-2000/2001 2004 Kampala: National Health Accounts

[B29] World Health Organization WHO Estimates for Country NHA Data 2011 http://www.who.int/nha/country/uga/en/

[B30] Ministry of Finance Planning and Economic Development: Background to the Budget FY 2004/2005 2004 Kampala: Ministry of Finance, Planning and Economic Development

[B31] Ministry of Finance Planning and Economic Development: Background to the Budget FY 2005/2006 2005 Kampala: Ministry of Finance, Planning and Economic Development

[B32] Ministry of Finance Planning and Economic Development: Background to the Budget FY 2006/2007 2006 Kampala: Ministry of Finance, Planning and Economic Development

[B33] Ministry of Finance Planning and Economic Development: Background to the Budget FY 2007/2008 2007 Kampala: Ministry of Finance, Planning and Economic Development

[B34] Ministry of Finance Planning and Economic Development: Annual Budget Performance Report FY 2007/2008 2008 Kampala: Ministry of Finance, Planning and Economic Development

[B35] Ministry of Finance Planning and Economic Development: Annual Budget Performance Report FY 2008/2009 2009 Kampala: Ministry of Finance, Planning and Economic Development

[B36] Ministry of Finance Planning and Economic Development: Approved Budget Estimates FY 2009/10 Volume 1 2009 Kampala: Central Government Votes

[B37] Ministry of Finance Planning and Economic Development: Approved Budget Estimates FY 2010/11 Volume 1 2010 Kampala: Central Government Votes

[B38] Ministry of Finance Planning and Economic Development: Background to the Budget FY 2011/2012 2011 Kampala: Ministry of Finance, Planning and Economic Development

[B39] Ministry of Health Annual Health Sector Performance Report FY 2003/2004 2004 Kampala: Ministry of Health

[B40] Ministry of Health Annual Health Sector Performance Report FY 2004/2005 2005 Kampala: Ministry of Health

[B41] Ministry of Health Annual Health Sector Performance Report FY 2006/2007 2007 Kampala: Ministry of Health

[B42] Ministry of Health Annual Health Sector Performance Report FY 2007/2008 2008 Kampala: Ministry of Health

[B43] Ministry of Health Annual Heath Sector Performance Report FY 2009/2010 2010 Kampala: Ministry of Health

[B44] Uganda Debt Network Uganda's Poverty Reduction Strategy Papers and Resource Allocation to the Health Sector 2004 Kampala: Uganda Debt Network

[B45] Organization for Economic Co-operation and Development Development Assistance Committee: Information note on the DAC Deflators 2011 http://www.oecd.org/investment/stats/informationnoteonthedacdeflators.htm

[B46] Ministry of Health Health Sector Strategic Plan III FY 2010/11-2014/15 2010 Kampala: Ministry of Health

[B47] Walford V A Review of Health Sector-wide Approaches in Africa 2007 London: HLSP Institute

[B48] Sundewall J Jönsson K Cheelo C Tomson G Stakeholder perceptions of aid coordination implementation in the Zambian health sector Health Policy 2010 95 2–3 122 128 20004996 10.1016/j.healthpol.2009.11.010

[B49] Chansa C Sundewall J McIntyre D Tomson G Forsberg BC Exploring SWAp's contribution to the efficient allocation and use of resources in the health sector in Zambia Health Policy Plan 2008 23 4 244 251 10.1093/heapol/czn013 18562459

[B50] Oomman N Bernstein M Rosenzweig S Following the Funding for HIV/AIDS: A Comparative Analysis of the Funding Practices of PEPFAR, GFATM, and World MAP in Mozambique, Uganda, and Zambia 2007 Washington D.C: Center for Global Development

[B51] African Forum and Network on Debt and Development Macroeconomic Policy Options in Sub–Saharan Africa: Linking Poverty Reduction Strategy Papers (PRSPs) and the Millennium Development Goals 2006 Harare: The Case of Uganda

[B52] Odaga J Lochoro P Budget ceilings and health in Uganda 2006 Kampala: Caritas Uganda

[B53] Rabkin M El-Sadr W De Cock K The impact of HIV scale-up on health systems: a priority research agenda J Acquir Immune Defic Syndr 2009 52 S6 S11 10.1097/QAI.0b013e3181bbcd69 19858943

[B54] Marchal B Cavalli A Kegels G Global health actors claim to support health system strengthening—is this reality or rhetoric PLoS Med 2009 6 4 e1000059 10.1371/journal.pmed.1000059 19399158 PMC2667637

[B55] Oomman N Bernstein M Rosenzweig S Seizing the Opportunity on AIDS and Health Systems 2008 Washington D.C: Center for Global Development

[B56] Ssengooba F Kiwanuka S Mbona N Rutebemberwa E Kirunda B Buregyeya E The Effects of Enhanced Availability of Funding from Global Health Initiatives on the Distribution, Retention and Motivation of Health Workers in Uganda 2009 Geneva: Alliance for Health Policy and Systems Research, World Health Organization

[B57] House of Commons Committee of Public Accounts Department for International Development: providing budget support to developing countries Report of Session 2007–08 2008 27 London: House of Commons

[B58] Consult J Phase II Evaluation of the Implementation of the Paris Declaration in Uganda Final Report 2011 Kampala: Government of the Republic of Uganda Office of the Prime Minister

[B59] MacKellar L Priorities in global assistance for health, AIDS and population Popul Dev Rev 2005 31 2 293 312 10.1111/j.1728-4457.2005.00066.x

[B60] Shiffman J Donor funding priorities for communicable disease control in the developing world Health Policy Plan 2006 21 6 411 420 10.1093/heapol/czl028 16984894

[B61] Esser DE Bench K Does global health funding respond to recipients’ needs? Comparing public and private donors’ allocations in 2005–2007 World Dev 2011 39 8 1271 1280 10.1016/j.worlddev.2010.12.005

[B62] Conway S Harmer A Spicer N External Review of the International Health Partnership+ Related Initiatives 2008 London: London School of Hygiene and Tropical Medicine

[B63] United States Government Global Health Initiative Strategy 2011 http://www.ghi.gov/resources/strategies/159150.htm

[B64] Atwood JB McPherson MP Natsios A Arrested development Foreign Affairs 2008 87 6 123 132

